# Fractional Er:YAG laser versus fractional CO2 laser in the treatment of immature and mature scars: a comparative randomized study

**DOI:** 10.1007/s00403-023-02764-6

**Published:** 2024-01-18

**Authors:** Mai Abdelraouf Osman, Ahmed Nazmy Kassab

**Affiliations:** 1 Dermatology and Laser at Dermatology Unit, Medical Laser Applications, National Institute of Laser Enhanced Sciences (NIlES) Cairo University, 25 Giza St, Giza, 11236 Egypt; 2 ENT and Laser at ENT Unit, Medical Laser Applications, National Institute of Laser Enhanced Sciences (NIlES) Cairo University, 25 Giza St, Giza, 11236 Egypt

**Keywords:** Fractional Er:YAG laser, Fractional CO2 laser, Scars, Dermoscopy

The optimum timing of laser treatment of scars is still debatable among experts. The purpose of this study was, therefore, to compare the efficacy and safety of 2,940-nm Er:YAG ablative fractional laser (AFL) versus 10,600-nm CO_2_ AFL for the treatment of immature and mature scars. Thirty two patients with post-traumatic and post-surgical immature (less than one year old) and mature scars (more than one year old) were enrolled in this study. All patients were divided into two groups according to the age of scars; group A with immature recent scars (n = 14) and group B with mature old scars (n = 18). Once more, group A and B were divided into two equal groups randomly to receive either Er:YAG or CO_2_ AFL. This study was approved by the Research Ethical Committee Of National Institute Of Laser Enhanced Sciences (NILES-EC-CU-23–3-5). Er:YAG AFL (Fotona Xs dynamics, Slovenia) was used with the following settings: hand piece PS01, short pulse mode (300 µs), energy fluence 800–1000 mJ/cm^2^, spot size 7 mm in diameter, frequency 5–7 Hz, pixel size (250–350 µ) and density (60–70 pixels). CO_2_ AFL (Smartxide DOT, DEKA, Italy) parameters were: power 10–15 W, dwell time 600 μs, spacing 700 μm, density level (3–5%) and smart stack, level 2. Er:YAG and CO_2_ laser sessions were conducted on monthly basis for 5 consecutive sessions with a follow-up visit at 3 months after the last session. Treatment efficacy was evaluated using clinical photographs, Vancouver Scar Scale (VSS), patient satisfaction, Dermatology Life Quality Index (DLQI), and dermoscopy at baseline, and at 3-month follow up. Regarding VSS, in Er:YAG AFL, group A showed significantly better appearance with respect to vascularity (0.71 ± 0.76, versus 1.67 ± 0.71, p = 0.021), pigmentation (0.43 ± 0.53, versus 1.22 ± 0.83, p = 0.046), scar height (0.43 ± 0.53 versus 1.44 ± 0.73, p = 0.008), and pliability (0.57 ± 0.79 versus 1.89 ± 1.27, p = 0.031), compared to group B. Likewise, in CO_2_ AFL, the vascularity (0.57 ± 0.79, versus 1.67 ± 0.71, p = 0.011), pigmentation (0.43 ± 0.53, versus 1.33 ± 0.87, p = 0.030), scar height (0.29 ± 0.49 versus 1.33 ± 0.71, p = 0.005), and pliability (0.29 ± 0.49 versus 1.67 ± 1.32, p = 0.021), were significantly better in group A compared to group B respectively.

In group A, comparing Er:YAG and CO_2_ AFL respectively, the difference in vascularity (0.71 ± 0.76, versus 0.43 ± 0.79, p = 0.502), pigmentation (0.43 ± 0.53, versus 0.14 ± 0.38, p = 0.271), scar height (0.43 ± 0.53 versus 0.29 ± 0.49, p = 0.611), and pliability (0.57 ± 0.79 versus 0.29 ± 0.49, p = 0.430) was not significant. In group B, the improvement in vascularity (1.67 ± 0.71, versus 1.44 ± 0.73, p = 0.384), pigmentation (1.22 ± 0.83, versus 1.11 ± 0.78, p = 0.270), scar height (1.44 ± 0.73 versus 1.11 ± 0.60, p = 0.764), and pliability (1.89 ± 1.27 versus 1.67 ± 1.32, p = 0.721) was almost the same in Er:YAG AFL compared to CO_2_ AFL and the difference between them was subtle. Patient satisfaction and DLQI score paralleled the physicians’ evaluations (Figs. 1 and 2). Dermoscopic images showed improvement of pigmentary and vascular components of immature and mature scars after both treatments (Figs. 3 and 4). AFL lasers induce microthermal treatment zones in a pixelated fashion through the epidermis and into the dermis at a regular pace, leaving zones of intact skin from which tissue regeneration commences [1]. The fractions of thermal injury elicit a cascade of various cytokines including heat shock protein, TGF-β and matrix metalloproteinase. This cascade is assumed to play a major role in normalization of the collagenesis–collagenolysis cycle [2]. The 2940 nm wavelength emitted by Er:YAG laser corresponds to the peak absorption coefficient of water, so nearly all of the energy is absorbed in the epidermis and papillary dermis. This leads to superficial tissue ablation and less underlying thermal damage and this accounts for the decreased collagen contraction and subsequently less dramatic clinical results compared to those obtained with CO_2_ laser [3]. This may interpret the superiority of CO_2_ laser over Er:YAG laser in the present study. It has been postulated that early laser intervention can display a trend towards a regenerative process and can modify the formation and distribution of collagen fibres similar to what is observed in normal scarless skin. Numerous studies reported the efficacy of AFL in scar management and suggested that early scar treatment can avoid undesirable bothersome scarring [4, 5]. Our data prove equivalent efficacy of Er:YAG and CO_2_ AFL in scar reduction. Furthermore, immature scars have better results than mature scars, intensifying the crucial role of early laser intervention in improving scar prognosis.

**Fig. 1:**
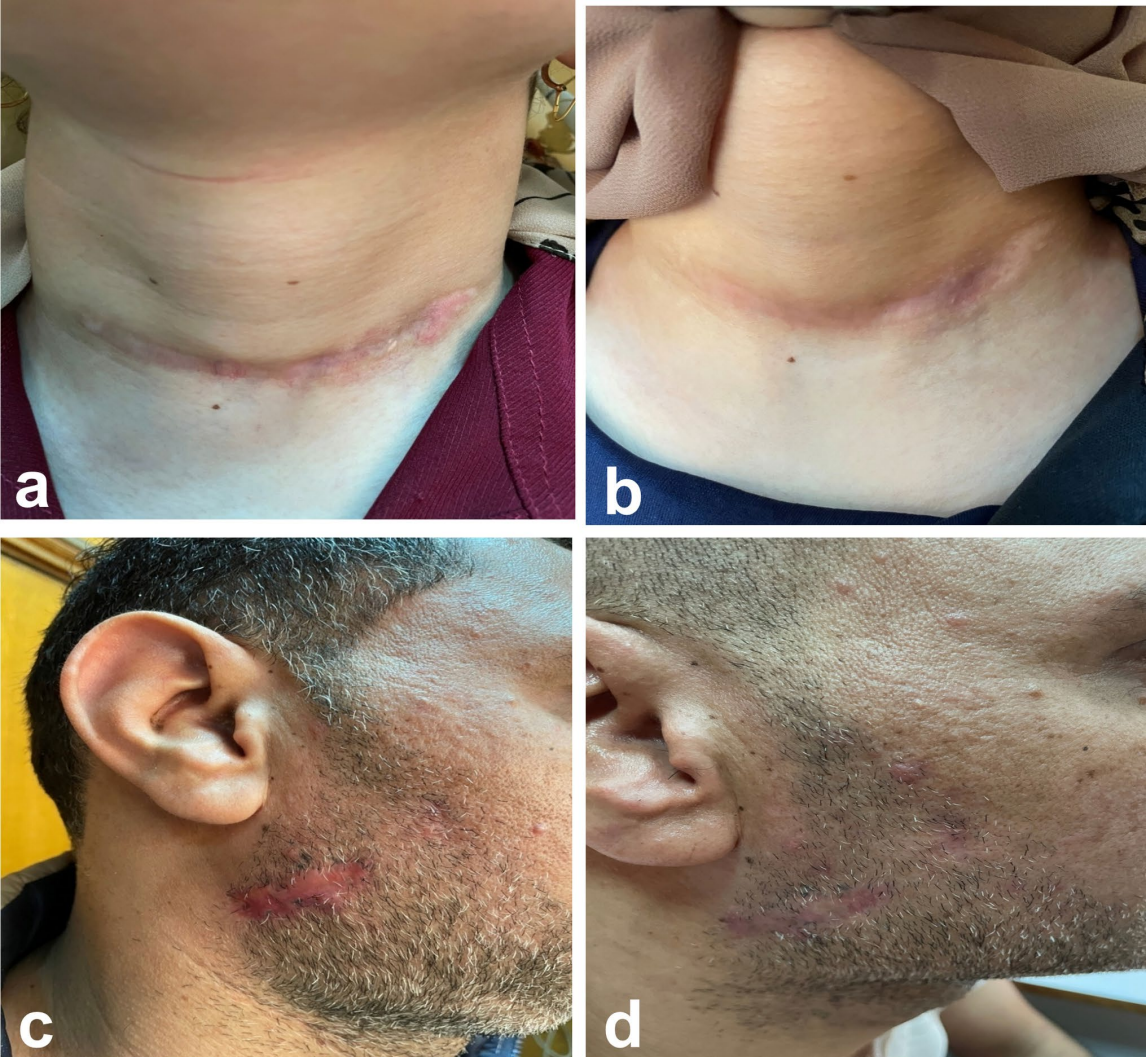
**a** Clinical picture of a 26- year-old female patient with an immature post-thyroidectomy scar on her neck before treatment, **b** At the 3-month follow-up after Er:YAG laser treatment. **c** Clinical picture of a 39-year- old male patient with an immature post-traumatic scar on his right cheek before treatment, **d** At the 3-month follow-up after CO2 laser treatment

**Fig. 2:**
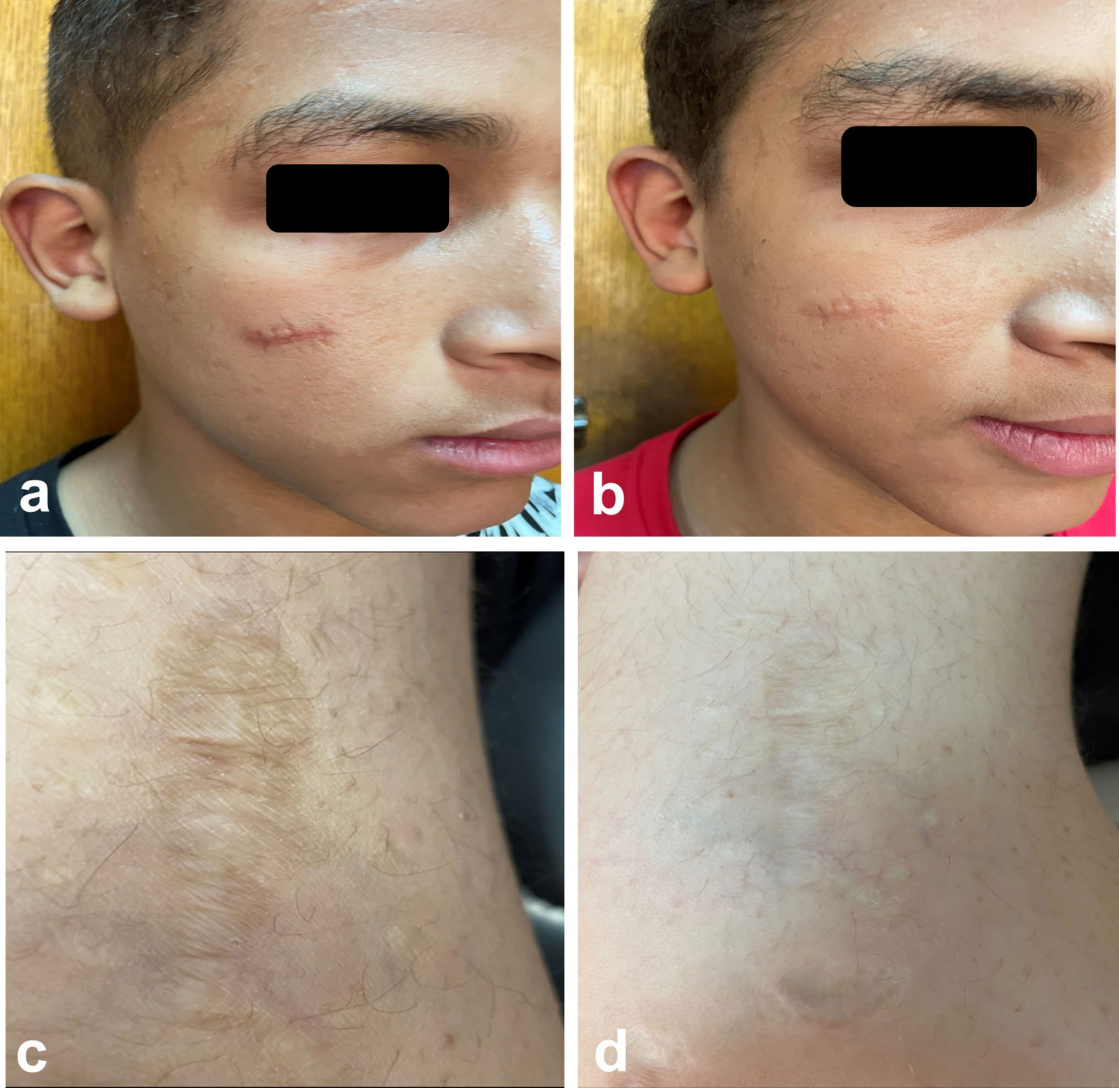
**a** Clinical picture of a 12- year-old male patient with a mature post-surgical scar on his right cheek before treatment, **b** At the 3-month follow-up after Er:YAG laser treatment. **c** Clinical picture of a 20-year-old male patient with a mature post-traumatic scar on his forearm before treatment, **d** At the 3-month follow-up after CO2 laser treatment

**Fig. 3:**
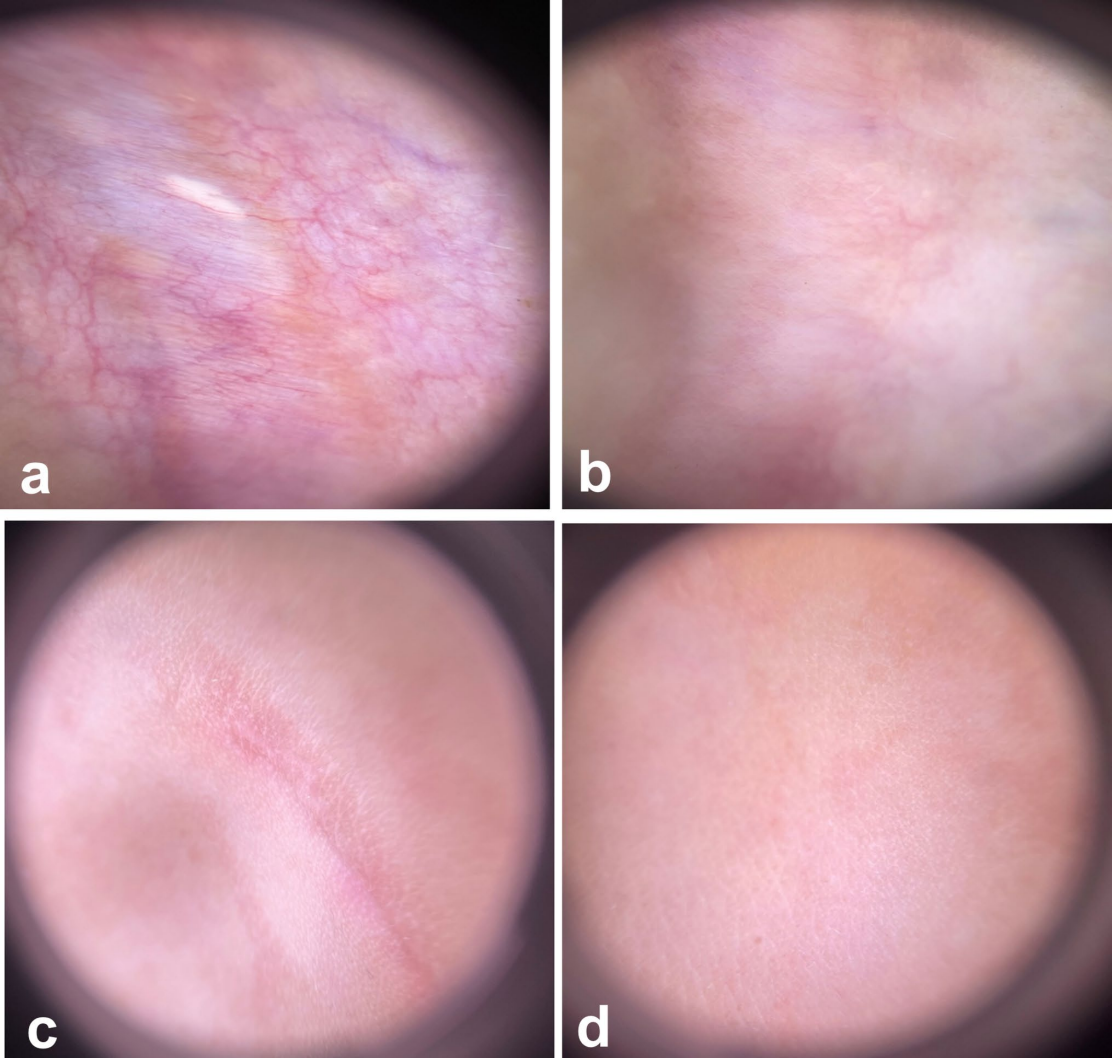
**a** Dermoscopic picture of an immature post-surgical scar showing vascular structures and arborizing vessels before treatment, **b** Marked improvement of vascularity at the 3-month follow-up after Er:YAG laser treatment. **c** Dermoscopic picture of an immature post-traumatic scar before treatment, **d** Near total resolution of the scar at the 3- month follow-up after CO2 laser treatment

**Fig. 4:**
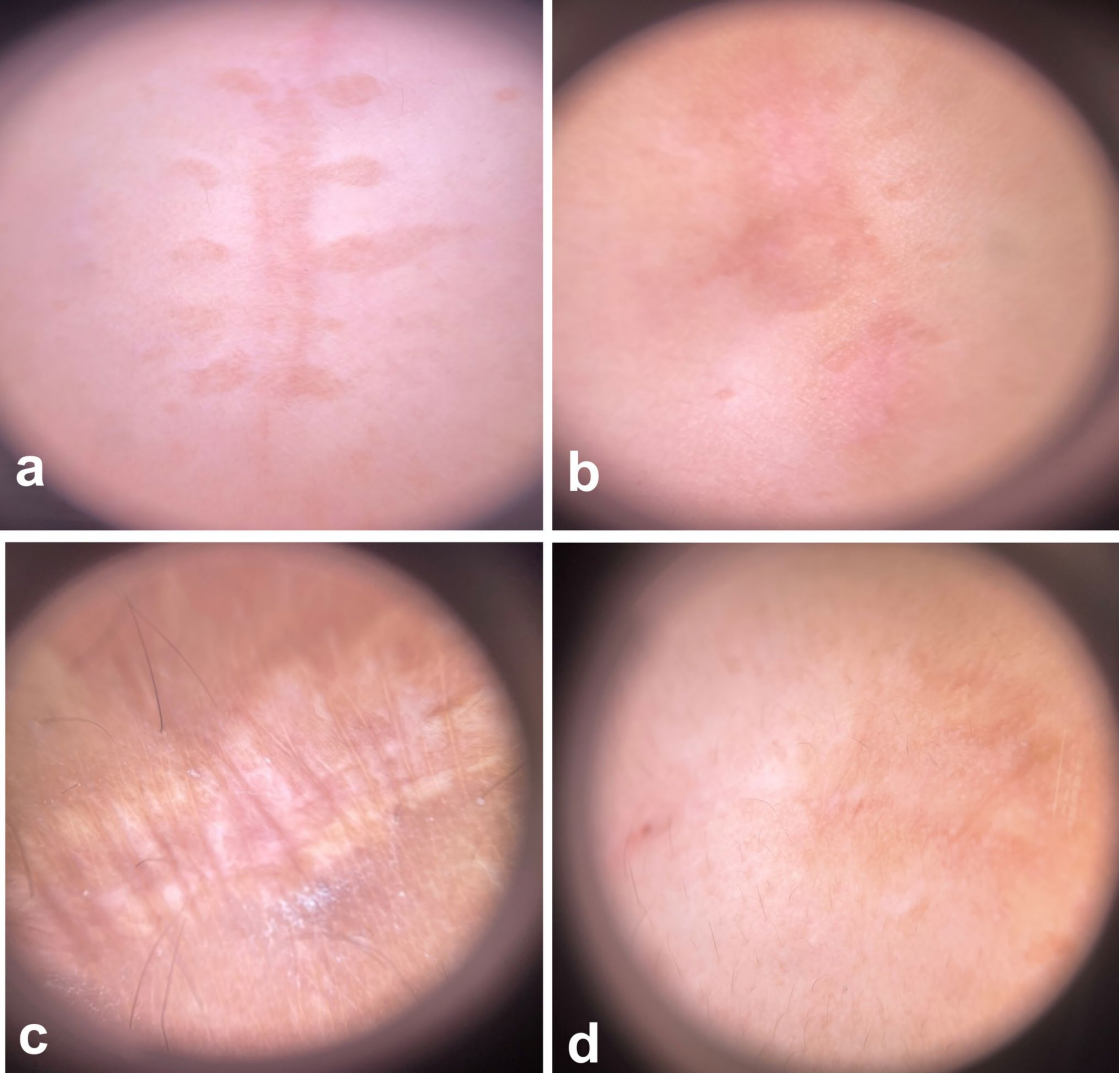
**a** Dermoscopic picture of a mature post-surgical scar before treatment, **b** Marked improvement of the scar at the 3-month follow-up after Er:YAG laser treatment. **c** Dermoscopic picture of a mature post-traumatic scar showing pigmentation and surface irregularities before treatment, **d** Near total resolution of the scar at the 3-month follow-up after CO2 laser treatment

## Data Availability

The data that support the findings of this study are available from the corresponding author upon reasonable request.

## References

[1] Willows BM, Ilyas M, Sharma A (2017). Laser in the management of burn scars. Burns.

[2] Helbig D, Mobius A, Simon JC, Paasch U (2011). Heat shock protein 70 expression patterns in dermal explants in response to ablative fractional photothermolysis, microneedle, or scalpel wounding. Wounds.

[3] ElAhmed HH, Steinhoff M (2021). Comparative appraisal with metaanalysis of erbium vs. CO2 lasers for atrophic acne scars. J Der Deutsche Dermatol Gesell.

[4] Kim SG, Kim EY, Kim YJ, Lee SI (2012). The efficacy and safety of ablative fractional resurfacing using a 2,940-Nm Er:YAG laser for traumatic scars in the early posttraumatic period. Arch Plast Surg.

[5] Sobanko JF, Vachiramon V, Rattanaumpawan P, Miller CJ (2015). Early postoperative single treatment ablative fractional lasing of mohs micrographic surgery facial scars: a split-scar, evaluator-blinded study. Lasers Surg Med.

